# Leveraging digital health initiatives to enhance the effectiveness, equity and responsiveness of health systems

**DOI:** 10.1136/bmjgh-2023-014626

**Published:** 2024-08-01

**Authors:** Sumit Kane

**Affiliations:** 1 Nossal Institute for Global Health, Melbourne School of Population and Global Health, The University of Melbourne, Melbourne, Victoria, Australia

**Keywords:** Health systems, Global Health, Health policy, Digital health

**Summary box**

- Digital health initiatives can and should help contribute to the achievement of health system goals.
- Digital initiatives should and have the potential to help improve equity, accountability, quality and efficiency of health systems.
- Digital initiatives can and should help subvert unscrupulous providers and enhance probity in health systems.
- Digital initiatives need to be rigorously evaluated against how they help achieve health system goals.
- This paper proposes a framework to evaluate and appraise current and new digital health initiatives.
- For digital health initiatives to fulfil their full potential, they need to be supported by robust governance, oversight and security arrangements.

## Introduction

 In August 2023, the G20 India presidency and the WHO announced a new Global Initiative on Digital Health (GIDH) at the Health Minister’s Meeting of the G20 Summit.^1^ The GIDH initiative will operate to support the implementation of the Global Strategy on Digital Health 2020–2025 and will focus on developing global standards, best practices and resources to fast-track digital health system transformation globally. The GIDH contends and there is emerging evidence to suggest that digital health interventions can help make health services:

1. Accessible, reliable and trustworthy.
2. Good quality.
3. Frictionless and seamless across levels and types of care.
4. Responsive and people centred.

There is also evidence to suggest that digital health interventions can improve health systems functioning more broadly through helping to:

1. Improve equity.
2. Improve transparency, accountability and probity.
3. Reduce effort and cost inefficiencies on the supply and demand sides.
4. Undermine and subvert unscrupulous providers.
5. Create demand for merit goods.
6. Improve the functioning of the healthcare market.

The question is how might one go about achieving this? What do countries need to keep in mind as they operationalise their digital health strategies? In the following section, drawing on global insights and taking a whole of health system perspective, and through using India as a case, I propose the outlines of and the logic underpinning a vision for national digital health initiatives to effectively leverage their ‘digital ecosystem’ to achieve health system goals.

## The digital ecosystem in India

India is chosen as a case as it helps illustrate, simultaneously, the constraints to and the opportunities for digital health interventions to fulfil their transformative potential. It is chosen, as in many ways, as the recent G20 India presidency, and its leadership in launching the GIDH^1^ suggests India’s work in digital health is being recognised globally. Finally, India is also chosen because it has in the last decade seen a vast variety of digital health initiatives—*BMJ Global Health*’s Special Issue on Digital Innovations for Community and Primary Health in India^2^ illustrates this. To begin with, some highlights of India’s burgeoning digital ecosystem are outlined—the situation in many low- and middle-income countries (LMICs) is similar or is rapidly becoming so.

- As of November 2022, India had more than 600 million smartphone users with internet connectivity,^3^ and Indians have embraced the possibilities offered by the digital world. The rapid and widespread adoption^4^ of the digital payment processes organised within the ambit of the national Unified Payments Interface,^5^ with transaction worth US$140 billion per month,^6^ exemplifies this.
- A national-level initiative, the Ayushman Bharat Digital Mission (ABDM),^7^ and its precursor, the National Digital Health Mission (NDHM), have established a complete ecosystem, a robust architectural frame and a suite of standards to offer digital solutions for improving healthcare in India.
- ABDM provides guidance to ensure interoperability, technological flexibility and independence. It sets out the compliance, technical integration, testing, scalability and minimum standards for data privacy protection. It has created the following core building blocks to enable the development and implementation of digital healthcare solutions:
  - A health ID—a unique health identifier for every individual in India.
  - A personal health record system that can draw from and be linked to multiple data sources.
  - A DigiDoctor—a unique identifier for every doctor in the country.
  - A health facility registry—a unique identifier and has standardised single point for all information for health facilities (hospitals, clinics, laboratories, pharmacies).
- The ABDM has also created a Sandbox^8^—an ecosystem that allows digital solutions to be developed and tested in a contained environment in compliance with ABDM standards. The Sandbox helps digital health initiatives to link with the building blocks of ABDM. By November 2023, one hundred and fifty such initiatives had graduated from the Sandbox and had achieved full compliance and integration.^9^

This digital ecosystem has spawned initiatives across public and private sectors and a range of care domains. A robust examination of what these initiatives are achieving is required—also required is an understanding (and ideally, an agreement) around what these initiatives should deliver for health systems, including but not limited to the Indian health system.

## What should the digital promise deliver for the Indian health system and for Indians?

*BMJ Global Health*’s Special Issue on Digital Innovations for Community and Primary Health in India and online supplemental appendix 1 provide a snapshot of the array of digital health initiatives in India; these either have information available in the public domain or are part of the ABDM’s Sandbox.^8^ These compilations reveal that initiatives are leveraging different aspects of the digital ecosystem to enhance users’ interactions with different parts of the health system. They also reveal how the focus, not unexpectedly, is disproportionately on the service provision and information systems. Few, if any, have been evaluated thoroughly; I argue that more than ever, the conditions are right for some of these initiatives to succeed. The question that follows is—what should this success look like? And what should these initiatives deliver for Indians?

**Figure 1:**
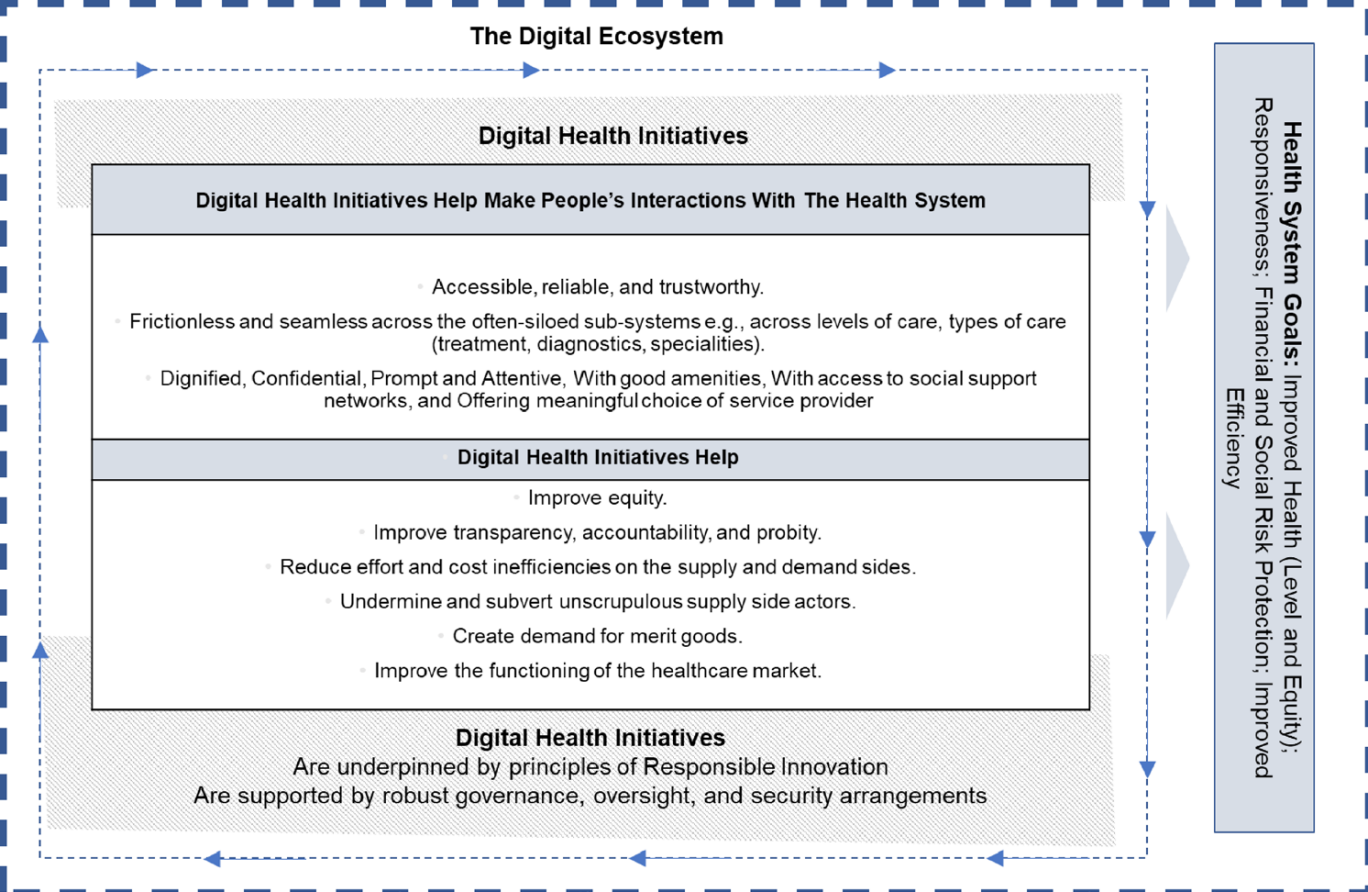
A vision and a guiding framework for digital health initiatives.

Next, with the 10 areas of contributions for digital health interventions articulated earlier as the backdrop, and drawing on global insights on digital health and taking a whole of health system perspective,^10^ the outlines of and the logic underpinning a vision for LMICs to leverage their ‘digital ecosystem’ to achieve many of the shared interests and health system goals are proposed. This vision is proposed also as a frame to evaluate and appraise current and new initiatives—figure 1 presents a diagrammatic representation of this guiding framework.

*Digital health initiatives should help contribute to* the achievement of health system-level goals of improved health (level and equity), responsiveness, financial and social risk protection and improved efficiency.

*Digital health initiatives should help make people’s interactions with* the health system accessible, reliable and trustworthy; frictionless and seamless across the often-siloed subsystems (eg, across levels of care, across types of care (treatment, diagnostics, specialties)); and responsive^11 12^ (ie, be dignified, confidential, prompt and attentive; with good amenities; with access to social support networks; and offering meaningful choice of providers). Evans *et al*’s^13^ human-centred design perspective is instructive.

*Digital health initiatives should help* improve equity; improve transparency, accountability and probity; reduce inefficiencies; undermine and subvert unscrupulous actors; create demand for merit goods; and improve the functioning of the healthcare market. Each of these imperatives requires careful and deliberate thought and action.

*Digital health initiatives should be underpinned by principles of responsible innovation*. While digital innovations offer wide-ranging benefits, they are criticised for focusing narrowly on technological and business aspects and ignoring important social and public good concerns.^14^ The concept of responsible innovation,^15^ which involves paying deliberate attention to the four-dimensional framework consisting of anticipation, reflexivity, inclusion and responsiveness, can help guide those mandated with the stewardship of the health system to ensure that the interests and goals outlined earlier always remain centre stage.

*Digital health initiatives need to be supported by robust governance, oversight and security arrangements*. The frequency and scale of healthcare data breaches has been growing rapidly globally.^16^ A comprehensive suite of robust measures, including but not limited to legislative measures,^17^ are thus necessary to ensure the integrity of digital data and to check hackers and fraudsters.

*Digital health initiatives must learn from global experiences and insights*. Three recent global works offer comprehensive lessons. The WHO’s ‘Digital Implementation Investment Guide (DIIG)’^18^ and ‘Recommendations on digital interventions for health system strengthening’^19^ offer insights to leverage digital technologies to tackle health system challenges. Similarly, the The Organisation For Economic Cooperation And Development (OECD) has developed a toolkit^20^ to guide those involved in designing, steering and delivering digital services. A recent systematic review^21^ adds that there is ‘urgent need for focused research aimed at generating high-quality evidence on the efficacy, user acceptability, and cost-effectiveness of mHealth interventions aimed toward health systems strengthening’.^21^

*These guidance documents also advise caution*. The pace of advancements in digital technologies is such that policy framework and systemic changes that are needed to ensure safety and equitable benefits have tended to lag to the detriment of public good. The DIIG’s cautionary note, ‘the lure of exciting new technologies and gadgets is ever present, but ultimately these technologies should be promoting health, keeping the world safe, and serving the vulnerable’,^18^ deserves full attention from those responsible for the stewardship of health systems.

## Conclusion

*Better stewardship is needed to achieve the full potential of digital health innovations*. This needs to be accompanied by robust, transparent and consistent mechanisms to steer digital health initiatives, to ensure that individually and collectively they all work towards the fulfilment of the vision articulated here. In the Indian context, the NDHM has the building blocks to help India see the vision through—similar mechanisms either exist in various stages of maturity across LMICs or need to be initiated.

## Supplementary material

10.1136/bmjgh-2023-014626 online supplemental file 1

## Data Availability

Data sharing not applicable as no datasets generated and/or analysed for this study.
