# Supplementary material for: Leveraging digital health initiatives to enhance the effectiveness, equity and responsiveness of health systems
Source: BMJ Glob Health. 2024 Aug 1;9(8):e014626. doi: 10.1136/bmjgh-2023-014626 (PMC11308869; doi:10.1136/bmjgh-2023-014626)
Supplement: online supplemental file 1 [file bmjgh-9-8-s001.pdf]

Appendix 1: A snapshot of the variety of digital health initiatives in India (Only includes those that have information available in the public domain or are part of the National Digital Health Mission's Sandbox).

#	Name	Status	Period Active	Targeted condition (if applicable)	Focus Area (By - Health Systems Building Block)	Public or Private Initiative	Weblink
1	Practo	Active	2008-present		Service Provision (teleconsultations, lab test booking), Information System (health locker, PHR, health-related information, search doctors by requirements, specialties, or symptoms), Supplies (home delivery of medicines and select FMCG products).	Private	https://www.practo.com/
2	Medibuddy	Active	2013-present		Service Provision (teleconsultations, lab test booking), Information System (health lockers, PHR, home delivery of medicines and select FMCG products, find doctors according to requirements, create ABHA health id, search doctors by specialties or symptoms).	Private	https://www.medibuddy.in/
3	e-Sanjeevani (2.0, HWC, OPD)	Active	2020-present		Service Provision (telemedicine, patient - to - doctor, doctor- to - doctor), Stewardship (hub and spoke model)	Public	https://esanjeevaniopd.in/
4	Docon	Active	2013-present		Information Systems (EMR, clinic experience digitization, teleconsultation appointment and health record management, reports and analytics, digital consultation, and practice management)	Private	https://docon.co.in/

#	Name	Status	Period Active	Targeted condition (if applicable)	Focus Area (By - Health Systems Building Block)	Public or Private Initiative	Weblink
5	DiNC, Kolar	Active	2017-present		Service Provision (telemedicine, patient - to - doctor, doctor- to - doctor), Information System (EHR, Non-Communicable Diseases (NCD) identification & care coordination, Mother-Child Care & coordination, Mental Healthcare, upward referrals and downward referrals, coordination with government health schemes.	Public Private Partnership (PPP)	https://www.tatasustainability.com/SocialAndHumanCapital/DigitalNerve
6	CPHC NCD Solution by Dell	Active	2017-present	NCDs	Devices (digital health tool for NCD screening), care coordination	PPP	https://www.dell.com/en-in/dt/
7	Karkinos	Active	2020-present	cancer	Stewardship (Cancer, care collaboration / care continuum, cancer knowledge network)	Private	https://www.karkinos.in/
8	Babylon Health (GP at Hand in the UK and Babyl in Rwanda)		(Babylon Health) 2013 - present (Babyl Rwanda) 2016-present		Information System (AI powered healthcare platform with virtual clinical operations for patients. Funded through a subscription-based based model, pay-as-you-go payments, centrally funded initiatives like NHS or as part of health insurance packages)	PPP	https://www.gpathand.nhs.uk/
9	TATA 1mg	Active	2015-present		Service Provision (teleconsultations, lab test booking), Information System (health locker, PHR, home delivery of medicines and select FMCG products, find doctors according to requirements, health-related information, search doctors by specialties or symptoms)	Private	https://www.1mg.com/?wpsrc=Google+Organic+Search

#	Name	Status	Period Active	Targeted condition (if applicable)	Focus Area (By - Health Systems Building Block)	Public or Private Initiative	Weblink
10	Bihar Command and Control Centre	Active	2021-present		Service Provision, Stewardship (Emergency health response)	Public	https://iccc.smartcities.gov.in/icc/city-details/
11	Services e-Health Assistance and Tele-consultation (SeHAT)	Active	2021- present (Home delivery of medicines 2022 onwards)		Service Provision (teleconsultation), Supplies (medicine delivery), Health Financing (insurance-linked)	Public	https://www.india.gov.in/
12	e-RaktKosh (Centralized Blood Bank Management System)	Active	2016-present		Information Systems (Blood related logistics (IT solution to connect, digitize and streamline the workflow of blood banks))	Public	https://www.eraktkosh.in/
13	Indian Railways Hospital Systems	Active	2020-present		Information Systems (HMIS system for railway hospitals)	Public	https://hmis.rcil.gov.in/AHIMS
14	Coronasafe	Active	2020-present	COVID-19	Information Systems (Toolkit of resources for addressing the COVID-19 pandemic, including capacity / patient management system for hospitals)	PPP	https://www.coronasafe.in/
15	Evolko	Active	2015-present		Service Provision (robotic triage, digital OPD management)	Private	https://evolko.com/
16	Jiyyo	Active	2017 - present		Service Provision (patient-to-doctor teleconsultation, search doctors by specialties or symptoms)	Private	https://www.jiyyo.com/
17	Mother and Child Tracking System MCTS / RCH Portal	Active	2009-present	RMNCH	Stewardship (Monitor and support the reproductive, maternal, new-born and child health (RMNCH) schemes /program delivery and reporting)	Public	https://rch.nhm.gov.in/RCH/
18	Integrated Disease Surveillance Programme (IDSP)	Active	2009-present		Stewardship	Public	https://idsp.mohfw.gov.in/

#	Name	Status	Period Active	Targeted condition (if applicable)	Focus Area (By - Health Systems Building Block)	Public or Private Initiative	Weblink
19	A-HMIS	Active			Information System (HMIS for health care delivery systems and patient care in AYUSH facilities)	Public	https://ehr.ayush.gov.in/home
20	e-Sushrut	Status Unclear	Unclear		Information System (HMIS, EHR)	Public	https://apps.gov.in/content/e-shushrut-hospital-management-information
21	OHumSky<br>(the OHumSky does not appear to be used anymore) the parent company is called OHUM Healthcare Solutions Pvt. Ltd)	Status Unclear	Unclear		Stewardship (Financial Management, Administrative Management and Clinical Management for hospitals. It also includes OhumBI, clinical and business analytics application guided by Evidence Based Medicine (EBM))	Private	https://www.ohumhealthcare.com/about-ohum/
22	Eka Care	Active	2020-present		Service Provision (PHR service)	Private	https://www.eka.care/
23	IHX	Active	2020-present		Information System (AI/ML based platform for healthcare management, Medical Risk Prediction, EHR)	Private	https://provider.ihx.in/
24	Kulcare	Active	2019-present		Information System (Appointment management, digital prescriptions, EHR), Service Provision (telemedicine)	Private	https://kulcare.com/
25	Central India E-Clinic	Status Unclear	Unclear		Information System (Practice management tools for doctors, health data management system for patients, appointment booking and management)	Private	https://www.ci-eclinic.com/
26	Tracelyfe (Racloop)	Active	2023-present		Information System (HMIS, PHR)	Private	https://tracelyfe.com/

#	Name	Status	Period Active	Targeted condition (if applicable)	Focus Area (By - Health Systems Building Block)	Public or Private Initiative	Weblink
27	Hodo	Active	2013-present		Information System (appointment booking system, online EMR system)	Private	https://hodo.io/
28	Redkenko Health	Active	2019-present		Health Financing (health financing, insurance)	Private	https://kenkohealth.in/
29	MocDoc (Yro Systems Private Limited)	Active	2012-present		Information System (HMIS, EMR, EHR)	Private	https://mocdoc.in/
30	S2 Infotech International Limited	Active	Began operations in 2005, unclear when health offerings started)		Information System (HMIS, EMR, EHR)	Private	https://www.s2iil.com/
31	National Health Authority Beneficiary Identification System (BIS) Portal for Ayushman Bharat	Active	2020-present		Stewardship, Information System, Service provision	Public	https://bis.pmjay.gov.in/BIS/mobileverify
32	National Health Authority Transaction Management System (TMS) Portal for Ayushman Bharat	Active	2020-present		Stewardship, Information Systems, Health Financing	Public	https://tms.pmjay.gov.in
33	TeCHO Gujarat (TeCHO Plus)	Status Unclear	2017-present		Stewardship (Improving coverage and quality of health services i.e, Android based application of ASHAs, MPW, CHOs; Web based portal for service providers and administrators at PHC/block/ district/ state level)	Public	https://techo.gujarat.gov.in/imtecho-ui/#!/

#	Name	Status	Period Active	Targeted condition (if applicable)	Focus Area (By - Health Systems Building Block)	Public or Private Initiative	Weblink
34	National Viral Hepatitis Control Program (NVHCP) NVHCP Portal	Active	2018-present	Viral hepatitis (acute and chronic)	Information System (Hepatitis information and management)	Public	https://nvhcp.mohfw.gov.in/
35	YoreCare (ADVANCE DIGITAL SOLUTIONS INDIA PRIVATE LIMITED	Active	2022-present		Information System (EHR), Supplies (home delivery of medicines)	Private	https://yore.care/
36	DRCFO	Status Unclear	Unclear		Unclear	Private	https://drcfo.in/
37	Dhanush Infotech - Uttarakhand Telemedicine	Status Unclear	2017 (pilot program launch) - present		Service Provision (telemedicine)	PPP	https://dhanushhealthcare.com/index.html
38	Employees' State Insurance Corporation (ESIC)	Active	1952 - present		Health Financing (health insurance)	Public	https://www.esic.gov.in/
39	Entro Labs IT Solutions Pvt Ltd	Status Unclear	2015-present		Service Provision (Technology partners for Digital Health implementation in the Public Health Domain for the Governments of Andhra Pradesh & Telangana)	PPP	https://www.entrolabs.com/
40	Caare	Active	2021 - present		Service Provision (Teleconsultation)	Private	https://caare.in/
41	ClinAlly	Active	2021-present		Information System (EHR , PHR, CDSS)	Private	https://www.clinally.com/
42	Apex Kidney Care Pvt Ltd	Active	2008 -present	Nephrological conditions	Stewardship (dialysis network)	Private	http://www.apexkidneycare.com/index.php
43	Connection Loops	Status Unclear	Unclear		Unclear	Private	https://www.connectionloops.com/
44	MyHealth	Active	2022-present		Information Systems (EHR, EMR), Services (appointment booking and management)	Private	https://www.myhealthcare.co/
45	eSAHAJ	Active	2022 - present		Information System (HMIS)	Private	https://esahaj.gov.in/

#	Name	Status	Period Active	Targeted condition (if applicable)	Focus Area (By - Health Systems Building Block)	Public or Private Initiative	Weblink
46	ANM AP Health EHR, National Health Mission, Andhra Pradesh	Active	Unclear		Information Systems (ABHA health ID creation, PHR)	Public	https://cfw.ap.nic.in/
47	7 Mantra For Health Universe	Status Unclear	2019 - present		Information Systems (EHR)	Private	https://manitechnest.com/
48	Integrated Health Management System, West Bengal Health and Family Welfare		2022 (year of integration with ABDM) - present		Information Systems (The IHMS dashboard web portal displays statistics pertaining to online OPD registraytions, e-prescriptions generated, Swasthya Ingit telemedicine)	Public	https://wbhealth.gov.in/
49	Farmako	Active	2019 - present		Information System (EHR), Supplies (medicine delivery)	Private	https://farmako.in/
50	Plus91 PHR	Active	2009 - present		Information Systems (PHR)	Private	https://www.plus91online.com/
51	Tatvacare (Digicare Healthcare Solutions Private Limited)	Active	2020 - present		Information Systems (practice management), Service Provision (telemedicine)	Private	https://www.tatvacare.in/
52	eHealth Kerala	Active	2017 - present		Service Provision (teleconsultations, appointment booking), Information Systems (creation of Aadhaar based health ID)	Public	https://ehealth.kerala.gov.in/
54	Bajaj Finserv Health for Doctors, Bajaj Finserv Health App	Active	2019 - present		Service Provision (Teleconsultations, appointment booking), Information Systems (creation of Aadhaar based health ID, EHR), Health Financing (insurance and healthcare EMI plans)	Private	https://doctors.bajajfinservhealth.in/
55	SRIT Healthcare	Active	1999 - present		Information Systems (EMR, HIS, medical imaging)	Private	http://sritindia.com/healthcare.php
56	HISP India		Unclear		Information Systems (Health informatics)	Private	https://hispindia.org/

#	Name	Status	Period Active	Targeted condition (if applicable)	Focus Area (By - Health Systems Building Block)	Public or Private Initiative	Weblink
57	MSTAR HEALTH E-GOVERNANCE PLATFORM	Status Unclear	Unclear		Service Provision (Telemedicine, Disease Surveillance, Medical Early Warning System, Medical / Disaster Advisory)	Private	http://www.hitachimgrmnet.com/technology-edge/product-approach/multi-completeness.html
58	Medsynapse RIS-PACS	Active	2003 - present		Stewardship (Teleradiology)	Private	https://www.medsynaptic.com/
59	NephroPlus	Active	2010 - present	Nephrological conditions	Information Systems (Dialysis network and mobile app for appointment management, teleconsultation, dialysis slot booking)	Private	https://nephroplus.com/
60	Raxa Health	Status Unclear	2011 - present		Information Systems (EHR, ABHA ID creation)	Private	https://www.raxa.com/
61	Portea	Active	2013 - present		Service Provision (Platform for managing appointment bookings, payment, family health ID and health records, at-home treatment Physiotherapy Services, care at home services, Elder Care Services, Postnatal Care)	Private	https://www.portea.com/
62	CoWIN	Active	2021 - present	COVID -19	Information Systems (vaccination management, COVID-19)	Public	https://www.cowin.gov.in/
63	Bahmni	Active	Unclear		Information Systems (EMR, HMIS)	Private	https://www.bahmni.org/
64	Doxper	Active	2015 - present		Information Systems (Clinical Workflow Management, Medical Informatics, EMR), Health Financing (Insurance)	Private	https://doxper.com/home/
65	Verraton Health	Status Unclear	2020 - present		Information Systems (HMIS, HRP)	Private	https://verratonhealth.in/
66	Athma SaaS	Active	Unclear		Information Systems (HMIS)	Private	https://athma.health/saas

#	Name	Status	Period Active	Targeted condition (if applicable)	Focus Area (By - Health Systems Building Block)	Public or Private Initiative	Weblink
67	Zuno	Active	2017 - present		Health Financing (Insurance)	Private	https://www.hizuno.com/
68	e-Manas: Karnataka Mental Healthcare Management System	Status Unclear	2020 - present	Mental health	Information Systems (digital registry of patient health records, statewide registry of mental health professionals, establishments, and patients)	Public	https://e-manas.karnataka.gov.in/#/
69	BMC-Mpower 1on1	Active	2020 - present	Mental health	Human Resources (Tele-counseling support from psychiatrists and clinical psychologists)	PPP	
70	MarSha Health Clinical Decision Support System	Status Unclear	2019 - present		Information Systems (CDSS)	Private	https://marshahealth.com/
71	Hello Health	Active	2009 - present		Service Provision (Health concierge service, rehabilitation services, teleconsultations)	Private	https://www.linkedin.com/company/hello-health/
72	eKincare	Active	2014-present		Service Provision (teleconsultations), Information Systems (health record management, health risk assessments, and preventive care reminders, end to end care coordination)	Private	https://www.ekincare.com/
73	Lybrate	Active	2014 - present		Service Provision (teleconsultations, in person and online appointment management)	Private	https://www.lybrate.com/
74	Apollo 24|7	Active	2020 - present		Service Provision (teleconsultations, in person and online appointment management, lab / diagnostics booking), Supplies (home delivery of prescription drugs and selected FMCG products)	Private	https://www.apollo247.com/AboutUs

#	Name	Status	Period Active	Targeted condition (if applicable)	Focus Area (By - Health Systems Building Block)	Public or Private Initiative	Weblink
75	Credihealth	Active	2013 - present		Service Provision (teleconsultation, in person and online appointment management)	Private	https://www.credihealth.com/
76	Pharmeasy	Active	2015 - present		Supplies (Selected FMCG products, prescription, and OTC drug home delivery)	Private	https://pharmeasy.in/
77	10bed ICU	Active	2021 - present		Information Systems (Remote management of critical care done viaTele ICU)	PPP	https://10bedicu.org/
78	Mera Aspataal" (My Hospital)	Active	2016 - present		Service Provision (Feedback collection portal for listed public hospitals)	Public	https://meraaspataal.nhp.gov.in/
79	Netmeds	Active	2015 - present		Supplies (Selected FMCG products, prescription, and OTC drug home delivery)	Private	https://www.netmeds.com/healthstore?
80	Qure AI	Active	2016 - present	Lung cancer, tuberculosis, heart failure	Service Provision (telehealth, AI backed imaging solutions, disease management)	Private	https://qure.ai/about-us/
81	Niramai	Active	2017 - present	Breast cancer	Devices (AI backed cancer screening device)	Private	https://www.niramai.com/
82	Khushi Baby	Active	2014 - present	RMNCH, NCD	Digital health census module for ASHA workers, Community Health Integrated Platform for NCDs, RMNCH, communicable diseases	Private	https://www.khushibaby.org/
83	Driefcase	Active	2016 - present		Information System (EHR)	Private	https://www.driefcase.com/
84	Tricog Health	Active	2014 - present		Stewardship (Remote management of critical care)	Private	https://www.tricog.com/
